# Draft Genome Sequence of an Obligate Psychrophilic Yeast, *Candida psychrophila* NRRL Y-17665^T^

**DOI:** 10.1128/genomeA.00851-17

**Published:** 2017-08-31

**Authors:** Broňa Brejová, Hana Lichancová, Filip Brázdovič, Andrea Cillingová, Martina Neboháčová, Ľubomír Tomáška, Tomáš Vinař, Jozef Nosek

**Affiliations:** a Departments of Computer Science and Applied Informatics, Faculty of Mathematics, Physics and Informatics, Comenius University in Bratislava, Bratislava, Slovak Republic; b Departments of Biochemistry and Genetics, Faculty of Natural Sciences, Comenius University in Bratislava, Bratislava, Slovak Republic

## Abstract

*Candida psychrophila* is an obligate psychrophilic yeast classified into the family *Debaryomycetaceae* (*Saccharomycotina*). Here, we report the draft genome sequence of the type strain, NRRL Y-17665. The genome sequence is 11.2 Mb long and codes for 5,827 predicted proteins.

## GENOME ANNOUNCEMENT

The yeast *Candida psychrophila* was originally isolated from penguin dung at Cape Royds, Ross Island (Antarctica) (1). Later studies showed its affiliation with the genus *Debaryomyces* belonging to the CTG clade of *Saccharomycotina* (2, 3). *C. psychrophila* is an obligate aerobe and psychrophile that does not grow at temperatures above 17°C (4). Its adaptation to cold environments is mediated by lipidome enrichment for unsaturated fatty acyl moieties (5, 6) and stress proteins, which are induced at a mild heat shock (25°C) (7).

In this work, the genome sequence of *C. psychrophila* was determined using Illumina HiSeq2500 technology. Genomic DNA was isolated from a clonal culture of the type strain grown overnight in YPD medium (1% [wt/vol] yeast extract, 2% [wt/vol] peptone, 2% [wt/vol] glucose) at 7°C with constant shaking. The DNA was extracted essentially as described previously (8) and purified using DNeasy minispin columns (Qiagen). The sequencing of a paired-end (2 × 101 nucleotides) TruSeq PCR-free DNA library was performed by Macrogen (South Korea). In total, 51,320,288 reads were generated. The low-quality ends of reads were trimmed by Trimmomatic (9), and the assembly was done by SPAdes version 3.9.1 (10) with K = 67. The mitochondrial genome and ribosomal DNA (rDNA) repeat were manually adjusted in Geneious version 5.6.6 (11). Contigs with coverage less than 10× or length less than 200 bp were discarded. The assembly was further polished with Pilon (12). The resulting assembly has a length of 11,241,723 bp in 193 contigs; the *N*_50_ is 487,949 bp, and the longest contig has a length of 1,179,685 bp. The GC content is 36.74%. Ten contigs terminate on one side with an array of telomeric repeats (TTATGAGGTGTCTGGATG). The sequence complementary to this motif was also found in the template domain of putative telomerase RNA (*TER1*).

To annotate the nuclear genome, protein-coding genes were predicted using ExonHunter (13) (with custom parameter training) and Augustus (14) (with model for *Debaryomyces hansenii*). ExonHunter reported 5,827 protein-coding genes, and Augustus 5,502. According to tRNAscan-SE version 1.3 (15), the genome contains 192 nuclear tRNAs, including 2 pseudogenes. The genome statistics are comparable to those of *D. hansenii*, which has a genome size of 12 Mb, with 6,284 predicted proteins in the UniProt proteome and 200 tRNAs. The BUSCO pipeline (16), comparing the ExonHunter gene set to 1,711 conserved single-copy orthologs in the order *Saccharomycetales*, reported 3 missing and 33 fragmented genes. Some of these are found in the Augustus predictions; combined ExonHunter and Augustus gene predictions are 98.9% complete using the BUSCO approach.

The genome sequence of *C. psychrophila* together with the genomes of *D. hansenii* (17) and *Debaryomyces fabryi* (18) provide a resource for comparative and functional studies, which may elucidate strategies for cold adaptation, including metabolic alterations, membrane composition, and stress responses. Moreover, the genome sequence would allow identification of cold-active enzymes suitable for biotechnology (19).

### Accession number(s).

This whole-genome shotgun sequencing project has been deposited in ENA/DDBJ/GenBank under the accession no. FYBW00000000. The version described in this article is the first version, FYBW01000000.
